# Neutrophil Extracellular Traps in Wound Healing of Diabetes: Mechanisms, Inducers, and Therapeutic Implications

**DOI:** 10.1155/mi/5525755

**Published:** 2026-06-08

**Authors:** Jiaojiao Xue, Zeyu Wang, Wenxiu Qi

**Affiliations:** ^1^ College of Integrated Traditional Chinese and Western Medicine, Changchun University of Chinese Medicine, Changchun, Jilin, China, ccucm.edu.cn; ^2^ Northeast Asia Research Institute of Traditional Chinese Medicine, Changchun University of Chinese Medicine, Changchun, Jilin, China, ccucm.edu.cn

**Keywords:** DFU, diabetic wound healing, DM, in vitro inducers, NETs, neutrophils

## Abstract

Wound healing damage, especially diabetic foot ulcer (DFU), is a serious complication of diabetes mellitus (DM). New evidence shows that neutrophil extracellular traps (NETs) are the key factor of this pathology. This review summarizes the literature from 2015 to 2024 to clarify the mechanism of NETs and its harmful effects on diabetes wounds. We have provided a detailed introduction to common in vitro NETs inducers such as phorbol 12‐myristate 13‐acetate (PMA), lipopolysaccharide (LPS), and calcium ionophore, as well as core protein markers such as citrullinated histone H3 (CitH3), myeloperoxidase (MPO), and neutrophil elastase (NE). Importantly, evidence from animal models and human patients suggests that sustained NETs maintain inflammation, hinder angiogenesis, and delay tissue repair. Finally, we explored promising clinical therapeutic strategies that promote healing by targeting NETs, such as DNase I degradation and inhibition of specific signaling pathways, highlighting the potential of NETs inhibition strategies in improving diabetic wound care.

## 1. Introduction

Diabetes mellitus (DM) is a chronic metabolic disease characterized by elevated blood sugar [1]. Severe hyperglycemia can cause symptoms such as polydipsia, polyuria, and weight loss, most commonly Type 1 diabetes mellitus (T1DM) and type 2 diabetes mellitus (T2DM) [2]. In addition, with the progress of DM, there are also many acute and chronic complications. One of the major chronic complications of DM is delayed wound healing, most of which occur in the feet, namely, diabetic foot; skin ulcers may also appear, and these wounds serve as an entry point for bacterial infection, which may lead to lower limb amputation and sepsis [3, 4].

Neutrophils are polymorphonuclear white blood cells that are also an important part of the human immune system’s defense against infection [5]. As the most abundant type of white blood cell in the human peripheral circulation, neutrophils are one of the cells that can be rapidly recruited to the site of infection, inflammation, or damage, are important immune cells for innate immunity, and are also the body’s first line of defense against pathogen invasion [6–8]. Studies have reported that in the early stages of infection, neutrophils directly phagocytally kill bacteria by secreting proteolytic enzymes, antimicrobial proteins, and reactive oxygen species (ROS) to resist the invasion of external pathogens [9–11].

In 2004, Brinkman first proposed neutrophil extracellular traps (NETs), whose unique and distinct death pathway from cell necrosis and apoptosis was named NETosis [12]. NETs are DNA–protein complexes characterized by a DNA backbone embedded with various protein components. The primary protein component is citrullinated histone H3 (CitH3), followed by granular proteins and peptides, including neutrophil elastase (NE) and myeloperoxidase (MPO), which are mainly composed of a specialized network structure that plays a crucial role in capturing and eliminating pathogens [12–14].

The formation process of NETs is that neutrophils are stimulated by inflammatory factors, activate the relevant signaling pathway through protein kinase C (PKC), leading to the production of large amounts of ROS; thus, the characteristic nuclear lobules of neutrophils disappear, and the chromatin enlarges, while the cytoplasmic membrane remains largely intact. Upon further stimulation, the cell membrane ruptures, releasing chromatin with antimicrobial proteins, unfolding to form NETs, which can effectively trap and kill bacteria [12, 15–17]. The release of NETs is a type of neutrophils immune response that is commonly used to prevent the proliferation of bacteria and eliminate pathogens; however, excessive production of NETs can also lead to tissue damage [18, 19]. Continuous evidence suggests that the sustained deposition of NETs may lead to pathological changes in various inflammatory diseases such as systemic lupus erythematosus, cystic fibrosis, deep vein thrombosis, rheumatoid arthritis, autoimmune diseases, and metabolic related diseases [20–27]. Therefore, elucidating the mechanism of action behind NETs has become a hot topic.

Reportedly, both in vitro and in vivo hyperglycemia can increase the release of NETs in neutrophils [28]. Studies have now shown that neutrophils in humans or laboratory animals may produce a large amount of superoxides and cytokines under hyperglycemic conditions, which triggers the formation of NETs [29–31]. According to recent research results, the wound healing of diabetes patients may be damaged by NETs formed by neutrophils [32, 33]. Based on the above studies, blocking the formation of NETs may be an effective strategy for diabetic wound healing. Current studies have shown that in a hyperglycemic environment in vivo, neutrophils experience oxidative stress and release cytokines such as IL‐6 and TNF‐α, which contribute to the formation of NETs in neutrophils [18, 32]. In diabetic patients or models, elevated blood glucose levels can stimulate the production of NETs and impede wound healing [28, 34, 35].

Chronic hyperglycemia acts as a direct stimulus, making neutrophils more susceptible to NETs; clinically, there is a dynamic association between NETs markers and glucose metabolism control. This suggests that hyperglycemia itself is one of the most direct drivers of NETs in the diabetic metabolic microenvironment [28]. Furthermore, a clinical and in vitro study found that neutrophils from diabetic patients are more prone to spontaneous NETs formation. High‐glucose stimulation can also induce NETs formation in neutrophils from healthy individuals. These observations confirm that both a hyperglycemic environment and high‐glucose stimulation can promote NETs formation. Further mechanistic studies indicate that high glucose drives NETs formation through NADPH oxidase (NOX)–dependent ROS signaling pathways, suggesting that oxidative stress is a key mechanistic node in the activation of neutrophils within the diabetic metabolic microenvironment [36].

Metabolomic analysis of neutrophils cultured in high‐glucose conditions and samples from patients with T2DM revealed that neutrophils undergo metabolic reprogramming under high‐glucose conditions, as evidenced by changes such as the accumulation of intermediates in the polyol pathway; furthermore, high‐glucose‐induced NETs formation is closely associated with ROS generated by NOX [9]. Glycolysis is the primary pathway by which neutrophils metabolize glucose [37]. Glycolysis is the primary pathway by which neutrophils metabolize glucose [38, 39]. However, NETs formation depends not only on glycolysis but also requires the participation of the pentose phosphate pathway (PPP) [40]. Upon stimulation by PMA and other agents, neutrophils reprogram their metabolism toward the PPP, accompanied by a significant increase in glucose‐6‐phosphate dehydrogenase (G6PD) activity. This enzyme facilitates the conversion of the glycolytic intermediate G6P into the PPP, generating NADPH, which powers NOX and thereby induces NETs formation. The use of 6‐aminonicotinamide completely blocks the release of superoxide and NETs, further indicating that the PPP plays a critical role in NETs formation [37, 41]. These findings reveal a new mechanism by which neutrophil metabolic reprogramming contributes to NETs release and provide a new perspective on the role of glucose metabolism in regulating NETs formation.

Metabolic changes in patients with T2DM promote the formation of advanced glycation end products (AGEs) [42]. These biochemical changes can lead to functional defects in innate immune cells such as neutrophils [43]. Meanwhile, existing studies have shown that the RAGE signaling pathway has been demonstrated to promote and amplify NETs formation. In diabetic conditions, the AGE burden in the body remains persistently elevated, leading to chronic activation of RAGE; conversely, inhibiting RAGE expression effectively blocks NETs formation and prevents neutrophils from rapidly generating NETs upon stimulation. Therefore, the AGE–RAGE axis may be one of the upstream amplifying mechanisms underlying the dysregulation of NETs in diabetes. However, whether AGEs directly induce NETs formation and whether this process is entirely dependent on the RAGE pathway requires further validation through additional original research [37, 44]. This indicates that high glucose levels, PPP, and AGEs can all activate NETs.

Current research suggests that NETs formed in diabetic conditions exhibit both quantitative and qualitative changes. Experiments using diabetic mouse models have shown a significant increase in the levels of the NETs biomarkers CitH3 and MPO in the serum of diabetic mice, indicating a substantial accumulation of NETs in the serum under diabetic conditions [45]. Studies have shown that, under hyperglycemic conditions, in vitro experiments reveal that high concentrations of glucose can significantly increase the production and release of NETs. Furthermore, levels of NE and double‐stranded DNA (dsDNA)—biomarkers of NETs—in the serum of patients with T2DM are higher than those in non‐diabetic control groups, indicating that hyperglycemia in diabetic patients increases the release of NETs and elevates the levels of NETosis‐related biomarkers [32, 33, 46, 47]. In T1DM, β‐cell death leads to the accumulation of neutrophils in the pancreas and is associated with increased NETs formation. In patients with T2DM, levels of dsDNA released from NETs are significantly elevated. NETs are induced by hyperglycemia, but their levels are also elevated in diabetic patients with strictly controlled blood glucose. It has therefore been found that hyperglycemia exacerbates the already elevated baseline levels of NETs in diabetic patients, thereby further increasing their harmful effects [48].

Diabetes not only promotes the excessive formation of NETs but, more importantly, causes qualitative changes in NETs, thereby shifting their function from a physiological defense mechanism to a pathological injury. The hyperglycemic and AGE‐rich environment associated with diabetes can lead to the citrullination and glycation of key NETs components, such as histones, MPO, and NE [49]. These modifications directly alter the charge, structure, and function of the proteins. Modified histones may be more toxic and cause greater damage to vascular endothelial cells [50]. The activity of MPO and NE may change, affecting their antimicrobial efficacy and pro‐inflammatory potential [51]. In addition, abnormalities in the structure and function of NETs have been observed. The NETs produced may have an abnormally dense or fragile physical structure, which affects their ability to effectively capture pathogens [52]. In addition, NETs with abnormal quality are cleared inefficiently and persist in tissues, serving as a source of chronic inflammation and autoantigens [49]. They are more likely to activate the immune system, trigger a type I interferon response, and directly damage endothelial cells, thereby accelerating the progression of diabetic vascular complications [53, 54]. Diabetes alters the microenvironment and cellular metabolism, leading to defects in the composition, structure, and function of NETs, thereby transforming them from defensive guardians into tissue‐destructive agents; this plays a key role in the pathogenesis of diabetes and its complications.

In diabetes, NETs‐related pathology stems not only from “increased production” but also from “impaired clearance”. Impaired clearance is a critical factor in the transition of NETs from a transient defense mechanism to a source of persistent damage, creating a “double whammy” of “overproduction and insufficient clearance” that accelerates disease progression. Impaired NETs clearance may have serious implications for organ damage and is implicated in many inflammatory diseases, such as T1DM or T2DM and diabetes‐related complications [55]. Under physiological conditions, NETs serve as a key mediator of antimicrobial defense. However, when NETs clearance fails, they become a driving force behind disease; excessive NETs release can lead to persistent chronic inflammation by sustaining cytokine secretion and oxidative stress [56, 57]. Abnormal degradation of NETs can also exacerbate endothelial damage, vascular dysfunction, thrombosis, and autoimmune responses [58, 59].

Defects in NETs clearance play a significant pathogenic role in diabetes. The underlying mechanisms involve macrophage dysfunction, persistent activation of the inflammatory response, and abnormalities in vascular endothelial cell function, all of which contribute to the development of diabetes and its complications.

### 1.1. Macrophage Dysfunction

In diabetic conditions, a hyperglycemic environment impairs macrophage function, leading to a reduced ability to phagocytose and degrade NETs [60]. A basic research study demonstrated that the metabolic state of macrophages determines their functional phenotype. In a hyperglycemic environment, GLUT1‐mediated glucose uptake increases, driving glycolysis and pro‐inflammatory (M1) polarization, while simultaneously impairing the alternative activation (M2) function associated with tissue repair. This provides a metabolic explanation for why the clearance function of macrophages is impaired in diabetes [61].

### 1.2. Persistent Activation of the Inflammatory Response

Similar to humans, diabetes involves inflammatory or metabolic components that predispose neutrophils in mice to form NETs [32]. Hyperglycemia promotes excessive NETs formation and release by neutrophils through enhanced oxidative stress and a persistent inflammatory state. In T1DM, elevated levels of circulating NETs‐associated proteins are already present in the early stages of the disease [62–64]. In T2DM biomarkers such as circulating free DNA and NE remain persistently elevated. Functionally, NETs impair wound healing, exacerbate neuropathy, nephropathy, and retinopathy, and promote tissue inflammation [65–67].

### 1.3. Abnormal Vascular Endothelial Function

Experimental and clinical studies of patients with diabetes have revealed that endothelial dysfunction is prevalent among this population, manifested as a reduced capacity to produce nitric oxide, which is closely associated with microvascular complications. In diabetes, endothelial dysfunction serves as a strong warning sign of invasive vascular complications [38]. Second, studies have shown that in diabetic conditions, impaired vascular endothelial function affects the clearance of NETs. By establishing a mouse model of diabetes with a NETs deficiency and conducting a series of tests, it was confirmed that vascular dysfunction does indeed exist; therefore, inhibiting NETs through two independent strategies can prevent the progression of vascular dysfunction in diabetic mice [33].

In summary, diabetes impairs NETs clearance through various mechanisms; the accumulation of NETs further exacerbates inflammatory responses and tissue damage, leading to progressive deterioration and increasing the incidence and severity of diabetic complications.

This paper aims to systematically sort out the structural composition of NETs, that is, the reticular structure formed by binding CitH3 and antimicrobial proteins (NE, MPO, etc.) with DNA as the backbone. Then, the method of constructing NETs in vitro is described, and common in vitro inducers (such as LPS, PMA, CI, IL‐8, IL‐1β, etc.) are summarized. Subsequently, the structure and function of the core markers in the formation of NETs (PAD4, NE, MPO, CitH3, DNA‐related proteins) were summarized. Further, this paper analyzes the molecular mechanisms involved in the development of NETs and diabetic foot ulcer (DFU) and other wound healing, as well as their regulation. It is hoped that NETs can be used as new therapeutic targets and provide new ideas and basis for broadening the clinical treatment of related diseases.

## 2. Methods

Literature published from 2015 to November 2024 was searched using PubMed, GeenMedical. Key search terms in both DFUs and NETs; diabetic wound healing and NETs, linking NETs to DM. Our inclusion criteria were research articles and the language was English. There are 28 articles on the induction methods of NETs in this review, 15 articles on DFU, and 11 articles on diabetic wound healing. The search included articles involving clinical and basic experimental research of the following types: (1) Clinical research on patients with DM, diabetic wound healing, and DFU and (2) In vitro experimental research (3) Research involving animal models. Initially, titles and abstracts were reviewed to identify pertinent articles, followed by a more comprehensive review.

### 2.1. NETs Networks and DM

#### 2.1.1. Establishment of NETs In Vitro

In vitro studies, the use of special inducers to stimulate neutrophils to induce NETs are the most common research method. Consequently, we have summarized four representative NETs inducers: (1) Phorbol 12‐myristate 13‐acetate (PMA); (2) Lipopolysaccharide (LPS); (3) Calcium ionophore (A23187); (4) Interleukin‐8 (IL‐8), Interleukin‐1 beta (IL‐1β), Cold‐inducible RNA‐binding protein (CIRP), Granulocyte colony‐stimulating factor (G‐CSF), and cancer‐associated fibroblasts, among others. These inducers are shown in Tables 1–4.

**Table 1:** Literature review of phorbol 12‐myristate 13‐acetate (PMA) in vitro methods for inducing NETs.

Ref.	Inducer	Cell types	Molding concentration	Animal models	Molding concentration	Diseases
[1]	PMA	Neutrophils	25 nM	NA	NA	NA
[2]	PMA	Neutrophils	100 nmol/L	C57Bl/6J mice	NA	DFU
[3]	PMA	Neutrophils	100 nM	db/db mice	50 mg/kg	DFU
[4]	PMA	PMNs	20 nM	NA	NA	NA
[5]	PMA	Neutrophils	25 nmol/L	G‐CSF^−/−^ and WT mice	NA	Rheumatoid arthritis
[6]	PMA	Neutrophils	100 nM	NA	NA	Diabetic wound healing
[7]	PMA	Neutrophils	100 nM	NA	NA	NA
[8]	PMA	Neutrophils	50 nM	WT and Panx1^−/−^ mice	NA	NA
[9]	PMA	Neutrophils	10 nM	Nonhuman primate model	NA	Sepsis
[10]	PMA	Neutrophils	25 nM	Albino adult Hartley guinea pigs	NA	Tuberculosis
[11]	PMA	Neutrophils	40 nM	NA	NA	NA
[12]	PMA; DEC	PMNs	600 nM	NA	NA	DFU
[13]	PMA	Neutrophils	0.5 μM	db/db mice	NA	DFU
[14]	PMA	Neutrophils	100 ng mL	STZ‐induced SD rats	60 mg kg	DFU
[15]	PMA	HL‐60 cells	100 nM	STZ‐induced C57BL/6 mice	40 mg kg	DFU
[16]	PMA	Neutrophils	100 nM	NA	NA	T2DM patients with purulent necrotizing lesions of the lower extremities
[17]	PMA	Human neutrophils	50 nM	Gulo^−/−^ mice	NA	Sepsis
[18]	PMA	Neutrophils	6.25 ng/mL	NA	NA	Pneumococcal disease
[19]	PMA	Neutrophils	20 nM	BALB/c mice	NA	Pneumonic tularemia

Abbreviations: ARDS, Acute respiratory distress syndrome; DEC, Diethylcarbamazine; DFU, Diabetic foot ulcers; PMNs, Polymorphonuclear cells; SD rats, Sprague Dawley rats; STZ, Streptozotocin.

**Table 2:** Literature review of lipopolysaccharide (LPS) in vitro methods for inducing NETs.

Ref.	Inducer	Cell types	Molding concentration	Animal models	Molding concentration	Diseases
[20]	LPS	Neutrophils	25 μg/mL	STZ‐induced ICR mice	60.0 mg/kg	DFU
[6]	LPS	Neutrophils	25 µg/mL	NA	NA	Diabetic wound healing
[7]	LPS	Neutrophils	1 μg/mL	NA	NA	NA
[21]	LPS	Neutrophils	10 µg/mL	NA	NA	Psoriasis

**Table 3:** Literature review of calcium ionophore in vitro methods for inducing NETs.

Ref.	Inducer	Cell types	Molding concentration	Animal models	Molding concentration	Diseases
[6]	CI	Neutrophils	4 µM	NA	NA	Diabetic wound healing
[8]	CI	Neutrophils	1 μM	WT and Panx1^−/−^ mice	NA	NA
[7]	CI	Neutrophils	4 μM	NA	NA	NA
[22]	CI	Primary mouse neutrophils	5 mmol/L	C75BL/6J mice and Lrg1^−/−^ mice	50 mmol/L	Cutaneous wound healing

Abbreviation: CI, calcium ionophore (A23187).

**Table 4:** Literature review of in vitro methods for the induction of NETs by IL‐8, IL‐1β, CIRP, G‐CSF, cancer‐associated fibroblasts, and other factors.

Ref.	Inducer	Cell types	Molding concentration	Animal models	Molding concentration	Diseases
[1]	IL‐8	Neutrophils	10 ng	NA	NA	NA
[5]	G‐CSF	Neutrophils	100 ng/mL	G‐CSF^−/−^ and WT mice	NA	Rheumatoid arthritis
[23]	IL‐1β	NA	NA	C57BL/6j mice; IL‐1β KO mice;	NA	Abdominal aortic aneurysm
[24]	FhAg	Ovine PMN	100 µg/mL	Sufolk sheep	NA	*Fasciola hepatica* causes liver fuke disease
[25]	Cancer‐associated fibroblasts	NA	NA	C57BL/6 mice	250 μg/mL	Tumors
[9]	Autologous platelet secretome	Neutrophils	NA	Nonhuman primate model	NA	Sepsis
[10]	*Mycobacterium tuberculosis*	NA	NA	Albino adult Hartley guinea pigs	Concentration of 1 × 10^10^ bacilli per mL	Tuberculosis
[26]	CIRP	Bone marrow‐derived neutrophils	5 µg/mL	WT and CIRP^−/−^ mice	NA	Sepsis
[27]	Light‐induced	Neutrophils	UVA (375 nm), blue (470 nm) and green (565 nm) light	NA	NA	SLE and dermatomyositis
[28]	Whole Wolbachia	Neutrophils	NA	WT or TLR6^−/−^ mice	NA	Onchocerciasis
[19]	*Francisella tularensis*	Neutrophils	1:20 (MOI)	BALB/c mice	NA	Pneumonic tularemia

Abbreviations: CIRP, cold‐inducible RNA, binding protein; FhAg, *Fasciola hepatica* soluble antigens; G‐CSF, granulocyte colony‐stimulating factor; IL‐1β, interleukin‐1 beta; IL‐8, interleukin‐8; SLE, systemic lupus erythematosus; WT, wild type.

#### 2.1.2. PMA

PMA is an activator of PKC and the most classic NOX‐dependent inducer of NETs formation [12, 52, 68, 69]. It belongs to the natural products of the phobolol ester class, which are terpenoids extracted from the croton genus. Its core mechanism is to activate PKC, which leads to the production of NOX‐dependent ROS in large quantities, which in turn triggers a series of intracellular events, which eventually cause chromatin to depolymerize and mix with granulin proteins and release them outside the cell—the classic “suicidal” NETosis [4, 12, 52, 70, 71]. In the study of autoimmune diseases, thrombosis, inflammation and other diseases, researchers often use PMA to stimulate neutrophils in healthy people or patients in vitro to simulate the formation of abnormal NETs in the body, and then study the role of NETs in the pathogenesis of diseases [72]. In patients with DM, the expression of PMA is significantly increased, so experts often use PMA to stimulate neutrophils in vitro, mimicking diabetes‐induced NETs [73].

Since neutrophils are terminally differentiated cells with a short lifespan, they are difficult to culture in vitro and are incapable of further proliferation [74]. This makes the study of its functioning a challenging issue. The human promyelocytic leukemia cell line HL‐60, when stimulated with all‐trans‐retinoic acid and other agents, exhibits granulocyte differentiation and has many functional characteristics with normal granulocytes [24, 75, 76]. Therefore, all‐trans‐retinoic acid and other drug‐induced HL‐60 cells offer a unique in vitro model for studying human myeloid cell differentiation [77]. After all‐trans‐retinoic acid induced HL‐60 cells to differentiate into granulocytes, NETs were produced following PMA stimulation (Table 1) [8].

#### 2.1.3. LPS

LPS, also known as endotoxin, is a major component of the cell wall of gram‐negative bacteria [78–80]. LPS is a pathogen‐associated molecular pattern inducer. LPS is widely known to be widely used in the establishment of inflammatory models because it can interact with TLR4, thereby activating the host defense system and initiating pro‐inflammatory mechanisms [80, 81]. In addition, LPS can also simulate bacterial infection and induce neutrophils to produce NETs, so it is often used as an inducer of NETs, but its potency is weaker than that of PMA. The core mechanism of LPS‐induced NETs formation begins with LPS activating the MyD88 signaling pathway through the TLR4 receptor complex, which initiates the Raf‐MEK‐ERK cascade to drive NOX2 complex assembly and ROS production [15, 82]. On the one hand, ROS promotes the release of NE and MPO from the particles and transports them to the nucleus, and on the other hand, they work together with these enzymes to degrade histones and destroy the nuclear membrane, leading to chromatin depolymerization [12]. Eventually, it leads to the mixed release of chromatin and intracellular proteins, completing the ROS‐dependent NETs formation process [52]. LPS can simulate a chronic, synergistic inflammatory environment, promote the formation of NETs and remove abnormalities in diseases such as sepsis, thrombosis, and systemic lupus erythematosus [83, 84]. Several studies have demonstrated that stimulation of neutrophils with 1–25 μg/mL of LPS is sufficient to produce excessive NETs in conditions such as DFU, diabetic wound healing, and psoriasis, thereby accelerating disease progression (Table 2) [85–88].

#### 2.1.4. Calcium Ionophore (A23187)

Calcium ionophore A23187 (CI) is an ionophore antibiotic that forms a dimeric complex with divalent cations such as Ca^2+^ [89]. As a common non‐classical, ROS‐independent inducer, it can directly mediate the influx of Ca^2+^, bypass the activation of cell surface receptors and NOX, and trigger a NOX‐independent NETs formation pathway [52]. The core mechanism is that in the CI‐induced NETs model, the intracellular Ca^2+^ concentration increases sharply, which activates calcium‐sensitive PAD4 and catalyzes CitH3, resulting in chromatin depolymerization. On the other hand, potassium ions (K^+^) are initiated by activating potassium channels. These two effects together lead to the release of NETs [90]. Different concentrations of CI can induce the formation of NETs in neutrophils, which often occur in skin wound healing and diabetic wound healing (Table 3) [86, 87, 91, 92].

### 2.2. IL‐8, IL‐1β, CIRP, G‐CSF, and Other Inducers

**Figure 1:**
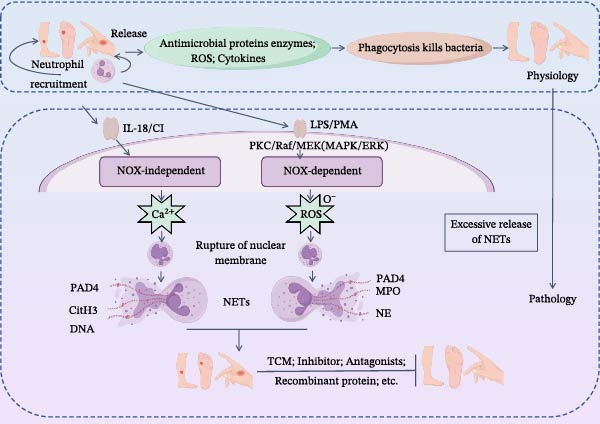
Mechanism of neutrophil extracellular trap formation in diabetic wound healing. Figure shows the induction and release mechanisms of NETs. In physiological processes, when the skin is damaged, the body first recruits neutrophils to reach the site of infection. Subsequently, neutrophils release substances such as antimicrobial proteases, ROS, and cytokines to deal with invading pathogens and kill bacteria through phagocytosis. Together, these mechanisms form the body’s defense system. In the pathological state, PMA/LPS and IL‐18/CI stimulated neutrophils can induce the formation of NETs through two pathways: NOX‐independent and NOX‐dependent, the former leading to nuclear membrane rupture and the release of NETs through Ca^2+^ signaling, and the latter triggering similar processes through ROS production. In turn, interventions may be made through TCM, inhibitors, antagonists, or recombinant proteins to stabilize the release of NETs and alleviate the disease process.

A study using a mouse model of abdominal aortic aneurysm suggests that IL‐1β‐induced NETs promoted the development of abdominal aortic aneurysm [93]. IL‐8, a chemokine and activator of neutrophils, attracts neutrophils to aggregate to sites of inflammation and induces the formation of NETs [12, 19]. In another study, stimulation of neutrophils with 10 ng IL‐8 for 45 min induced the formation of NETs [12]. Currently, a study provides evidence that *fasciola hepatica* soluble antigen induces innate immune responses and intracellular ROS production in sheep Polymorphonuclear leukocytes (PMNs), as well as the formation of NETs, in vitro and in vitro [94]. Similarly, in another study, experts treated isolated mouse bone marrow PMNs with cancer‐associated fibroblasts and found that it can induce the formation of NETs within tumors as well as in blood and bone marrow throughout the body, and these NETs are driven by ROS mediated pathways [95]. Additionally, the results of a mechanistic study demonstrated that NETs were induced using autologous platelet secretome and PMA. Notably, compared to autologous platelet secretome, PMA was observed to induce a greater DNA surface area in a sepsis model. Furthermore, it was found that treatment with activated protein C effectively inhibited the formation of PMA‐induced NETs [96]. Interestingly, in the guinea pig model, *Mycobacterium tuberculosis* was able to induce NETs in vivo, and it was observed that NETs could be detected as early as 30 min after inoculation [97]. Recent experimental data clearly show that CIRP can induce the formation of NETs in the lungs of mice with sepsis and may serve as a new inducer of NETs [98]. Interestingly, the experts’ findings suggest that blue and long‐wave ultraviolet light induces NETs in patients with autoimmune deficiency diseases such as systemic lupus erythematosus and dermatomyositis, an association offers a strategy for further exploration of photosensitizing diseases and the development of new therapeutic drugs [99]. Strikingly, for the first time in human onchocerciasis, it has been demonstrated that the wolbachia endosymbiont induces the formation of NETs [100]. In a mouse model of collagen‐induced arthritis, G‐CSF induces excessive bone marrow neutrophils, which may lead to the production of NETs, accelerate joint inflammation, and ultimately lead to bone erosion, so blocking this pathological process is a potential strategy for the treatment of rheumatoid arthritis [74] (Table 4, Figure 1). The studies mentioned above indicate that PMA, LPS and CI are the most commonly used strong inducers of NETs. Additionally, the efficiency and mechanisms of NETs induction vary depending on the applied stimuli.

In diabetes research, different NETs inducers are used, with the aim of simulating the different pathophysiological environments in the disease in vitro, thus revealing the specific role of NETs in diabetes and its complications. PMA, as a potent agonist of PKC, induces strong NETs by activating the NOX‐dependent pathway, and is commonly used to assess the intrinsic response of diabetic hyperglycemic status to neutrophils. Studies have shown that diabetic mice‐derived neutrophils are significantly more responsive to PMA, which provides a mechanistic basis for understanding persistent non‐infectious inflammation (such as atherosclerosis) in diabetic patients [32]. LPS functions through TLR4, and its core value is to mimic chronic low‐grade inflammation (such as metabolic endotoxemia) and infection susceptibility states common in diabetes. LPS often acts as a “priming” signal to make neutrophils more sensitive to subsequent stimuli, which explains why diabetic patients are more prone to uncontrolled systemic inflammatory responses in the face of infection in animal models, with NETs being the key effector [101]. The calcium ion carrier (A23187) directly mediates Ca^2+^ influx and bypasses NOX to activate PAD4, thereby revealing a NETosis pathway that does not depend on hyperglycemia and oxidative stress. This pathway is particularly important in diabetes‐related thrombosis and ischemia‐reperfusion injury, as inhibition of PAD4 has been shown to be effective in reducing thrombotic burden in animal models of diabetes, validating the translational medical value of this pathway [102]. In summary, PMA is used to define the intrinsic NETosis potential of cells, LPS is used to study the cross‐dialog between infection and chronic inflammation, and calcium ion carriers are used to dissect the pathogenic mechanism of NOX‐dependent, all of which jointly advance our systematic understanding of the role of NETs in the complex inflammatory network of diabetes.

### 2.3. Pathological Characteristics and Core Markers of NETs

Since its report, two mechanisms of NETs formation have been described: classic suicidal NETosis and vital NETosis [103]. Suicidal NETosis is primarily induced by PMA [103]. Correspondingly, in suicidal NETosis, ROS is produced by activating the Raf/MEK/ERK signaling pathway and NOX, which ultimately leads to the death of neutrophils through cell membrane lysis and the release of NETs [15, 104, 105]. In the Raf/MEK/ERK signaling pathway, experts found that PAD4‐dependent histone nitination can induce DNA deconcentration, resulting in proteins such as MPO and NE being included in intracytoplasmic particles [90, 103]. In vital NETosis, DNA bursts out of the nucleus, crosses the cytoplasm, binds to the plasma membrane, transports the DNA outside the cells, forms NETs without damaging the cell membrane, and maintains the integrity of neutrophils [104]. Currently, NETs‐DNA, CitH3, MPO, PAD4, and NE, as well as other NETs‐binding proteins, play a crucial role in studying the antimicrobial properties of NETs [105].

#### 2.3.1. MPO Enhances the Bactericidal Capacity of NETs and Promotes the Formation of NETs

MPO is a heme‐containing peroxidase that is highly expressed in various inflammatory cells such as neutrophils [106]. During the formation of NETs, MPO, a major granule protein, is released from aniline blue granules in the cytoplasm and is converted to hypochlorous acid by the enzyme catalase, which subsequently activates NE [12, 87, 107]. Subsequently, NE and MPO translocate into the nucleus, where they collaborate to promote chromatin decondensation [22, 108]. Meanwhile, MPO is the main component of post‐translational modification of self‐antigens contained in NETs, which is related to chromatin deaggregation in the process of NETs [87, 107, 109, 110]. The main function of MPO is that in the event of an infection, its bactericidal properties help destroy pathogens [111]. Studies have also shown that MPO is a major component of the immune response to infection [112]. In addition, MPO may stabilize the structure of NETs through the generation of covalent cross‐links in proteins [113, 114].

#### 2.3.2. Peptidyl Arginine Deiminase 4 (PAD4) Catalyzes Citrullination of Histone

PAD4, a member of the peptidyl arginine deiminases (PAD) family, is a nuclease that is highly expressed in peripheral blood neutrophils and has classical nuclear localization signals [115–118]. It has been shown in the literature that PAD4 is indispensable for the formation of NETs and that its involvement in NETs‐mediated bacterial capture and elimination [19, 50, 119, 120]. The important role of PAD4 is that inducers such as PMA, LPS, and CI activate PAD4, which is involved in the formation of NETs in various diseases [120]. Chromatin decondensation and nuclear depolymerization in NETs are thought to be mediated by post‐translational modifications of histones, whereas in the study found that PAD4 reduces histone positive charge and thus mediates histone citrullination [50, 68, 120].

#### 2.3.3. CitH3 Is a Marker of NETs Formation and Participates in NETs‐Mediated Biological Processes

Histone can be modified by a variety of modifications, including phosphorylation, acetylation, glycosylation, methylation, ubiquitination, and citrulination, and CitH3 is a citrullinated modified molecule of histone H3 that can be detected in the nucleus [50, 121–123]. Importantly, CitH3 is considered the gold standard for the formation of NETs, serving as the core component and main detection index [124]. The principle of the modification is that PAD4 catalyzes the citrullination of histone H3. This process diminishes the electrostatic interactions between histones and DNA, resulting in chromatin decondensation [107, 120]. Subsequently, the nucleus of neutrophils loses its lobulated structure, and the nuclear membrane dissolves, leading to the fusion of cytoplasm and nucleoplasm. Finally, as the cell membrane of neutrophils ruptures, the release of NETs occurs [125]. Therefore, CitH3 in neutrophils is frequently regarded as a biomarker for NETs, and its content can reflect the level of NETs production [93, 126].

#### 2.3.4. NETs‐DNA Constitutes the Reticular Skeleton of NETs

The DNA component of NETs (NETs‐DNA) is a potent antimicrobial agent whose antimicrobial activity is primarily attributed to its ability to sequester surface‐bound cations, disrupt the integrity of cell membranes, and lyse bacterial cells [105, 127]. NETs consist mainly of chromatin DNA filaments encapsulated by granulin as the main backbone structure [34, 128]. Furthermore, it has been suggested that during the formation of NETs, a significant amount of host double‐stranded DNA fibers are arranged into thicker aggregates to form a meshwork [104, 129, 130]. And notably, most of the DNA found in NETs originates from the nucleus [84]. Additionally, the binding of CitH3 to cellular free DNA is considered a major hallmark of NETs formation [125].

#### 2.3.5. NE Facilitates the Diffusion of NETs and Participates in the Sterilization Process

NE, as an alkaline serine protease, is stored in neutrophils blue‐philic granules to assist neutrophils in migrating to the foci of inflammation and exert their antimicrobial activity [131, 132]. The primary function of NE is to alter the cytoskeleton of neutrophils, releasing it from basophils into the cytoplasm. Within the cytoplasm, NE first binds to and degrades f‐actin to inhibit actin dynamics, then transports to the nucleus where it degrades specific histones, promoting chromatin decondensation [12, 51]. Subsequently, NE synergizes with MPO and NOX‐generated bactericidal peptides and ROS to assist phagocytosis and lysosomal degradation, thereby killing and degrading invading pathogenic microorganisms under controlled conditions. [12, 51, 132, 133].

### 2.4. DFU

DFU are one of the most serious chronic complications of diabetes [134]. It is a disease characterized by high morbidity, disability, mortality, and significant physical and mental suffering [35, 135]. Common clinical features include infection, ulceration, and gangrene; in severe cases, amputation may be necessary [136]. DFU typically occur on the sole of the foot, where microvascular complications cause local tissue hypoxia or ischemia, leading to the formation of ulcerated surfaces [137]. Due to the accumulation of large numbers of neutrophils in DFU wounds and persistent inflammation, delayed healing of DFU occurs [18, 138]. A series of studies in recent years suggests that NETs produced by neutrophils can delay the wound healing process [5, 18, 32]. Although the relationship between DFU and NETs in delaying wound healing has been demonstrated to varying degrees, the precise molecular mechanisms by which NETs delay diabetic wound healing remain unclear.

#### 2.4.1. Mechanisms of NETs‐Induced Damage in DFU

##### 2.4.1.1. Chronic Inflammation and Impaired Angiogenesis

A study not only confirmed that NETs induce NLRP3 inflammasome formation and activation in macrophages via TLR/NF‐κB and ROS/TXNIP pathways but also demonstrated that these NETs can be eliminated by DNase I, thereby regulating inflammatory responses and offering a therapeutic approach for diabetic wound healing [139].

Similarly, another in vitro study demonstrated that in diabetic mouse models, NETs may induce endothelial‐to‐mesenchymal transition via the Hippo‐YAP pathway, thereby inhibiting angiogenesis, and confirmed that NETs delay wound healing [140]. In addition, this study involving both humans and mice demonstrated that components of NETs were enriched in unhealed DFU in humans. In mice, examinations, including biopsy microscopy, revealed the presence of NETs in excised wounds. There is also evidence that specific PKC βII inhibitors not only stimulate angiogenesis in diabetic patients but also prevent the excessive NETs from occurring in neutrophils, thereby accelerating wound healing in diabetic mice and offers important insights for the development of new therapeutic approaches for diabetic patients [85]. Recent studies have demonstrated that milk fat globule epidermal growth factor VIII, acting as an inhibitor of the NLRP3 inflammasome‐NETs axis, can resolve wound inflammation, reduce NETs accumulation, promote angiogenesis, and accelerate wound healing [35]. Furthermore, the results of a randomized controlled trial indicate that negative pressure wound therapy (NPWT) enhances blood flow perfusion by promoting angiogenesis; it reduces the formation of neutrophil‐induced NETs by disrupting their structure and altering the bacterial microenvironment, and lowers the proportion of M1 macrophages in the wound, ultimately accelerating wound healing in patients with DFU) compared to traditional moist dressings, NPWT offers significant advantages [141].

#### 2.4.2. Therapeutic Strategies Targeting NETs

##### 2.4.2.1. Inhibitor

Interestingly, an animal study revealed that disulfiram suppresses the release of NETs in STZ‐induced diabetic mouse models via the NLRP3/Caspase‐1/GSDMD pathway, thereby promoting the healing of DFU [142]. In a clinical study, the researchers observed a significant upregulation of CitH3, recognized as the gold standard for NETs, in samples from patients with DFU; at the same time, the researcher demonstrated that the degradation of NETs occurred following the administration of DNase I in a rat model of DFU, resulting in enhanced wound healing [124]. A study in mice and humans found that neutrophils isolated from the blood of patients with DFU exhibited increased spontaneous NETs formation and impaired NETs‐inducing capacity. In in vivo experiments, PAD4 inhibition with Cl‐amidine reduced the levels of citrullinated histones (CitH3/4) and dsDNA in mouse neutrophils stimulated by the bacterial toxin ionomycin. Furthermore, pretreatment with Cl‐amidine reduced PAD4 activity in wound extracts from diabetic mice, thereby decreasing NETs formation and restoring normal wound healing in these mice. These findings suggest that inhibiting NETs may be a potential therapeutic strategy for accelerating wound healing in DFU patients [33].

##### 2.4.2.2. Small Molecules, Biomarkers, or Targets

NETs first gained recognition for their active role in bacterial killing processes [12]. However, we reported in a recent study that miRNA‐26b‐5p inhibits NETs by targeting MMP‐8 to accelerate diabetic wound healing [143]. Additionally, data from one study suggest that the activation of TREM1 promotes the recruitment of FOXM1+ neutrophils and reduces the formation of NETs, thereby accelerating wound healing in diabetic mice [144]. Similarly, a study discovered elevated levels of NETs in patients with DFU and in LepRdb/db diabetic mice; and for the first time, researchers found that hydrogen sulfide promotes the healing of diabetic wounds by inhibiting the release of NETs [73]. Another study employed a combined approach of bioinformatics and machine learning to identify Fibroblast Growth Factor Receptor 2 (FGFR2) as a potential biomarker for NETs‐driven inflammation in the DFU microenvironment. Analysis of animal studies and clinical trials involving DFU patients revealed reduced expression of FGFR2 in diabetic wounds. Furthermore, the modulatory effects of the existing drugs canagliflozin and gliquidone on FGFR2 suggest that targeting the FGFR2 pathway may represent a future therapeutic strategy for improving DFU outcomes [145]. Other studies have found that, in DFU patients receiving multidisciplinary treatment, NETs‐specific markers are negatively correlated with wound healing, and CitH3 is a potential biomarker. Observations indicate that the microenvironment surrounding foot ulcers promotes the release of NETs by neutrophils, thereby impairing wound healing. Therefore, targeting NETs may alleviate impaired wound healing [146].

##### 2.4.2.3. Other Drugs With Therapeutic Potential

An analysis of another study indicated that diethylcarbamazepine delayed and reduced NETs in polymorphonuclear neutrophils in healthy individuals and patients with T2DM, thereby potentially promoting healing of foot ulcers in diabetic patients, highlighting the therapeutic potential for DFU [137]. Similarly, in a mouse model of diabetes, treatment with gonadotropin‐releasing hormone (GnRH) antagonists accelerated wound healing induced by GnRH agonists; Similarly, in vitro experiments demonstrated that GnRH antagonists reduced PMA‐induced NETs. These findings confirm that GnRH antagonist therapy suppresses NETs and promotes wound healing in diabetic conditions, potentially offering a novel therapeutic strategy for refractory DFU [147]. In addition, recent evidence emphasizes that mercury‐containing preparations inhibit the formation of NETs in both diabetic mice and humans by suppressing the ERK1/2 pathway, and these findings demonstrate the therapeutic efficacy of mercury‐containing preparations in ameliorating DFU, thus providing a scientific rationale for their clinical application [18], as shown in Table 5.

**Table 5:** The main study of neutrophil extracellular traps in diabetic foot ulcers.

Group title	Ref.	Year	Model/patients	Drug/targets	Biomarkers	Conclusion
Chronic inflammation and impaired angiogenesis	[14]	2019	DFU patients; SD rats	DNase I	ROS; NLRP3; IL‐1β; pro‐IL‐1β; CD66b; CD68; CitH3; ASC; active caspase‐1; AIM2, NLRC4	Elimination of NETs by a topical DNase I treatment promotes wound healing by regulating the NLRP3 inflammasome and inflammatory cell infiltration
	[29]	2023	WT or Padi^−/−^ Mice; DFU patients; HUVECs	NETs inhibitors	CitH3; cfDNA; MPO‐DNA complex; PAD4; Snail‐1; CD31	NETosis delays diabetic wound healing by inducing EndMT via the Hippo‐YAP pathway
	[20]	2018	Male ICR mice	PKC βII	PKCβII; Akt; eNOS; VEGF; CitH3; DNA	Ruboxistaurin accelerates wound healing in diabetic mice by preventing neutrophils from undergoing excessive NETosis prompting the angiogenesis process to reverse impaired EPC functions
	[30]	2020	Healthy controls, diabetic patients, and DFU patients; WT(C57BL/6) and Mfge8^−/−^ mice	MFG‐E8	IL‐1β; IL‐18; TNF‐α; MPO; CitH3; DNA; active caspase‐1; MFG‐E8; HMGB‐1	MFG‐E8 improves angiogenesis and accelerates wound closure
	[31]	2023	DFU patients	NPWT	NE; MPO	Negative pressure wound therapy is superior to conventional moist dressings in wound bed preparation prior to STSG surgery for patients with chronic DFUs
Inhibitor	[32]	2022	Wild‐type (WT), Gsdmd^−/−^, Aim2^−/−^, Nlrp3^−/−^, Casp‐1^−/−^, Casp‐11^−/−^ BALB/c mice	Disulfiram	PAD4; NLRP3; caspase‐1; GSDMD; MPO; CitH3	Disulfiram could inhibit NETs‐mediated diabetic foot ulcer healing impairment by suppressing the NLRP3/Caspase‐1/GSDMD pathway
	[33]	2020	Healthy and diabetic patients with DFU; DFUs in diabetic rats	DNase	CitH3; ROS; DNA; NLRP3; IL‐1β	Eliminating NETs from activated neutrophils, significantly improve the wound healing process in rats with diabetes via lowering the release of IL‐1β thus, uncovering a novel approach to treat DFUs
	[2]	2016	Control subjects without diabetes; patients with diabetes; patients with DFUs; C57BL/6J mice	PAD4 inhibitor Cl‐amidine	CitH3/4; PAD4; MPO; nucleus; chromatin; NE; dsDNA; NGAL, proteinase‐3, and lactoferrin	This study in mice and humans shows that NETosis can delay wound healing in patients with diabetes
Small molecules, biomarkers, or targets	[34]	2024	Diabetic patients; C57BL/6 J mice	miRNA‐26b‐5p	MMP‐8; CitH3; MPO	Exosomal miRNA‐26b‐5p suppresses NETs by targeting MMP‐8 to promote diabetic wound healing
	[13]	2022	Db/db mice; human DFU specimens, and human neutrophils	FOXM1	ROS; CitH3; Keratin 5; TREM1; Ly6G; CXCL2, CXCL3, CXCL8; CCL2; STAT3	TREM1 promoted the recruitment of FOXM1^+^ neutrophils and reversed effects of diabetes and promoted wound healing in vivo
	[35]	2019	LepR^db/db^; LepR^m+/db^; 14 diabetic foot patients	H2S	PAD4; CitH3; dsDNA; MPO; NE	H2S attenuates NETosis and primes diabetic wound to heal through blockage of ROS‐mediated MAPK ERK1/2 and p38 activation
	[36]	2025	DFU patients; C57BL/6J mice	Canagliflozin; Gliquidone	FGFR2	FGFR2 may be a biomarker and therapeutic target associated with NETs in DFU
	[37]	2020	DFU patients; diabetic patients without foot ulcers and 60 healthy controls	NA	CitH3; dsDNA; MPO; NE Circulating levels of cfDNA, nucleosomes	NET‐specific markers negatively correlated with wound healing in DFU patients, and CitH3 is a potential marker
Other drugs with therapeutic potential	[12]	2020	Healthy adults and DM2 patients	DEC	NE	DEC delays and decreases the NETosis by PMNs from DM2 people
	[15]	2020	C57BL/6 mice; HL‐60 cell; DFU patients	GnRH	PAD4; CitH3; p‐ERK and p‐P38; GnRH receptor	GnRH antagonist treatment inhibited NETosis and promoted diabetic wound healing
	[3]	2024	Male C57BL/6J mice; C57BLKS/Db/db diabetic mice	MCP	PAD4; CitH3; MPO; IL‐8; ROS	MCP can mitigate the release of NETs, likely by suppressing the ERK1/2 signaling pathway

*Note:* LepR^db/db^, lacking leptin receptor 101; LepR^m+/db^, their normoglycemic control mice; Akt; Protein kinase B.

Abbreviations: DEC, diethylcarbamazine; DM2, type 2 diabetes mellitus; EndMT, endothelialmesenchymal transition; eNOS, endothelial nitric oxide synthase; EPCs, endothelial progenitor cells; FGFR2, fibroblast growth factor receptor 2; GnRH, gonadotropin‐releasing hormone; H_2_S, hydrogen sulfide; HSFs, primary human skin fibroblasts; MFG‐E8, milk fat globule epidermal growth factor VIII; MMP‐9‐IN‐1, MMP‐9 inhibition; NPWT, negative pressure wound therapy; PKC βII, protein kinase C βII; SD, Sprague Dawley rats; VEGF, vascular endothelial growth factor.

In summary, NETs are present in the wounds of diabetic patients and diabetic model rats or mice. Inhibiting NETs can significantly enhance wound healing in both diabetic rat models and diabetic patients.

### 2.5. Diabetic Wound Healing

Delayed wound healing is a significant complication of diabetes and has garnered increasing attention and research from experts and scholars in recent years [148]. Among the 500 million people worldwide with DM, approximately 25% suffer from skin ulcers that are prone to recurrent infections and difficult to heal [4]. Wound healing consists of four successive and overlapping phases: hemostasis, inflammation, proliferation, and remodeling [149]. When any one of the four stages is impaired, wound healing is delayed. Upon tissue injury, neutrophils are the first to arrive at the wound site, rapidly activating to release NETs that suppress bacteria and pathogens, thereby initiating a specialized defense mechanism known as NETosis [149]. NETosis, a distinct form of cell death, impacts wound healing in diabetic patients, and studies have shown that high glucose stimulates the excessive production of NETs, which in turn hinders the healing process of diabetic wounds [147].

#### 2.5.1. Mechanisms of NETs‐Induced Damage in Diabetic Wound Healing

##### 2.5.1.1. Chronic Inflammation and Impaired Angiogenesis

A study has demonstrated that the absence of leucine‐rich α‐2‐glycoprotein 1 leads to impaired inflammatory responses, epithelial regeneration, and angiogenesis, resulting in delayed wound healing. In diabetic mice with Lrg1 gene knockout, reduced NETs activity was observed; it is hypothesized that targeted regulation of LRG1 may serve as an effective strategy to inhibit excessive NETs formation, thereby accelerating wound healing in diabetic patients [91]. Interestingly, experts discovered that NETs containing eDNA formed at corneal epithelial wounds in diabetic mice. Subsequently, they found that DNase I could eliminate eDNA and promote healing of corneal epithelial wounds in diabetic mice, while also improving inflammation resolution, corneal nerve fiber regeneration, and mechanical sensation recovery. This may represent a promising potential therapeutic strategy for diabetic keratopathy [150]. A recent clinical cohort study documented increased wound inflammation in diabetic patients with elevated levels of NETs in wound exudate; Subsequently, the team developed a novel NETs clearance bio‐based hydrogel microsphere “microcage”‐mPDA‐PEI@GelMA that is beneficial for wound repair by combining methylacrylic gelatin (GelMA) hydrogel microspheres with cationic polyethyleneimine (PEI)‐functionalized mesoporous dopamine (mPDA). In STZ‐induced diabetic mouse models, it can achieve the effect of non‐contact removal of NETs and ROS on the surface of nanomaterials, effectively reducing the inflammatory response of wounds in mice and promoting wound healing [151]. M@M‐Ag‐Sil‐MA (a light‐curable methylacryloylated silk fibroin protein hydrogel (Sil‐MA) system co‐loaded with metformin‐supported mesoporous silica microspheres (MET@ MSNs) and silver nanoparticles (Ag NPs)). The study found that M@M‐Ag‐Sil‐MA inhibited the formation of NETs and reduced the release of pro‐inflammatory factors induced by NE, MPO, and NETs in a diabetic mouse model. This system promotes diabetic wound healing by modulating the immune microenvironment, accelerating tissue repair and angiogenesis [152]. A study revealed that Ro 106‐9920 promotes skin cell regeneration and enhances angiogenesis by inhibiting the formation of NETs, and alleviates inflammatory responses by blocking the activation of the NLRP3 inflammasome, thereby promoting wound healing in diabetic patients [153]. A study developed a positively charged protein‐based adhesive bioshield (PCPAB). This material binds to the free DNA in NETs, thereby inhibiting NETs‐induced inflammatory cytokine expression and eliminating the inhibitory effect of NETs on the focal adhesion signaling pathway. Consequently, it significantly enhances epithelial cell migration, endothelial cell proliferation, and angiogenesis, ultimately accelerating the process of diabetic wound healing [154].

##### 2.5.1.2. Impaired Macrophage Polarization and Phagocytic Capacity

A research team has developed a photodynamic hydrogel (OD/PBA‐PEI/Ce6@ALDA‐1 Hydrogel) that activates ALDH2. Through immune‐vascular synergy, this hydrogel effectively reduces the formation of NETs and alleviates chronic inflammation, while promoting the polarization of macrophages from the pro‐inflammatory M1 phenotype to the reparative M2 phenotype, thereby promoting angiogenesis and improving wound healing in diabetic patients [155]. Furthermore, in one study, pine pollen polysaccharides (PPPS) promoted wound healing in diabetes by mitigating high‐glucose‐induced damage to fibroblasts, keratinocytes, and human umbilical vein endothelial cells (HUVECs), degrading NETs, and inhibiting M1 macrophage polarization. Similarly, experimental data from diabetic mouse models indicate that PPPS can improve skin wound closure rates, reduce scar width, and increase the density of CD31‐positive blood vessels at the wound site. This provides evidence that PPPS can improve diabetic wound healing [156]. A study has revealed that, in both cell co‐culture systems and STZ‐induced diabetic mouse wound models, the excessive formation of NETs leads to impaired phagocytic function in macrophages, which in turn results in delayed wound healing in mice. Specifically, inhibiting the excessive formation of NETs or targeting the PI3K/Rac1 pathway effectively restored phagocytic capacity in macrophages, suggesting that these pathways may serve as potential therapeutic targets for promoting wound healing in diabetes [157].

#### 2.5.2. Therapeutic Strategies for NETs in Diabetic Wound Healing

##### 2.5.2.1. Inhibitor

Data from another study suggest that high blood glucose levels stimulate neutrophils to overproduce NETs, while DNase I degrades NETs and accelerates wound healing in both diabetic mice and diabetic patients [32]. A study has demonstrated that the IL‐8/MMP‐9 axis causes delayed wound healing in type 2 diabetes through NETs‐mediated neutrophil‐fibroblast interactions, Subsequently, in a model of NETs‐stimulated human skin fibroblasts (HSFs), treatment with DNase I or MMP‐9‐IN‐1 was found to block the NETs/IL‐8/MMP‐9/HSFs vicious cycle, suggesting these molecules may serve as potential therapeutic targets for treating delayed wound healing in T2DM [67]. In another study, researchers developed a hierarchically assembled hydrogel dressing composed of quaternized chitosan and HOCl‐responsive nanogels, which locally delivers platelet‐derived growth factor‐BB (PDGF‐BB) and DNase I with distinct kinetic profiles. In a skin wound model in diabetic mice, it was demonstrated that this hierarchical hydrogel promotes neutrophil recruitment, enhances endothelial cell migration, clears excess NETs, and prevents infection, thereby accelerating wound healing [158].

##### 2.5.2.2. Small Molecules, Biomarkers, or Targets

A study clarified that hypoxic pretreated MSC‐derived small extracellular vesicles (sEVs) effectively promote diabetic wound healing and reduce overproduction of NETs by targeting miR‐17‐5p. Further experimental studies have shown that this effect inhibits the formation of NETs by targeting the TLR4/ROS/MAPK pathway, providing a scientific basis for targeted therapy of NETs [159]. Data from another study demonstrated that reducing the release of epithelial cell‐derived high‐mobility group box 1 (HMGB1) inhibited the formation of NETs, accelerated cutaneous wound healing in mice, and decreased the risk of skin tumorigenesis [160]. Researchers have demonstrated in wound healing models using diabetic mice and HSFs that NETs trigger endoplasmic reticulum stress by activating the IRE1α/XBP1 signaling pathway, thereby inducing ferroptosis in fibroblasts and leading to impaired wound healing in diabetes. Notably, treatment with KIRA6 effectively alleviates NETs‐induced cellular damage and significantly promotes wound healing [45].

##### 2.5.2.3. Other Drugs With Therapeutic Potential

Furthermore, in a diabetic mouse model of *Staphylococcus aureus* skin wound infection, neutralizing alpha toxin decreased the production of NETs and accelerated the healing of infected wounds in diabetic mice [161]. Similar results were reported in another study indicating that insulin may enhance diabetic wound healing by inhibiting the excessive formation of NETs induced by LPS and PMA [86]. Notably, studies have shown that in diabetic patients, clarithromycin may enhance antimicrobial defense and improve wound healing by upregulating LL‐37 within the structure of NETs [162]. A recent study developed a multifunctional hydrogel composed of phenylboronic acid‐grafted quaternized soy protein isolate, sodium alginate, and HCM (BQSA‐HCM). In vitro and in vivo studies confirmed that this hydrogel system promotes diabetic wound healing by upregulating BMAL1 expression and inhibiting NETs formation via the PGE2/EP2‐EP4/BMAL1 signaling pathway. These findings suggest a new therapeutic strategy for diabetic wound healing [163]. Supplementation with vitamin D3/omega‐3 unsaturated fatty acids has been shown to inhibit the excessive production of NETs by PMA‐stimulated neutrophils in whole blood samples from patients with T2DM, which may positively impact wound healing in individuals with T2DM [164].

In brief, these data above provide exciting evidence for therapeutic strategies for diabetic wound healing (as shown in Table 6).

**Table 6:** The main study of neutrophil extracellular traps in diabetic wound healing.

Group title	Ref.	Year	Model/patients	Drug/targets	Biomarkers	Conclusion
Chronic inflammation and impaired angiogenesis	[22]	2020	Adults between 21 and 90 years old with T2DM; C75BL/6J mice; HL‐60; HDMECs; wild‐type and Lrg1^−/−^ mice	LRG1	LRG1; CitH3; AKT, p‐AKT	LRG1 promotes wound closure in diabetes patients by inhibiting excessive NETosis
	[38]	2020	Male C57BL/6 mice	DNase I	ROS; MPO; NE; CitH3; PAD4; Ly6G; F4/80; CD206; Inos; CD86; TNF‐α, MCP‐1; IL‐12; IL‐10; eDNA	DNase I improves wound healing in diabetes by inhibiting the formation of NETs
	[39]	2024	22 burn patients and 12 diabetic wounds patients; C57BL/6 mice	mPDA‐PEI@GelMA	CitH3‐DNA complexes; cfDNA; cfmiRNA; ROS; IL‐6, MPO; CitH3; TNF‐𝛼; IL‐1β; IFN‐γ	mPDA‐PEI@GelMA reduces inflammatory reaction and accelerates wound healing of diabetes by clearing NETs
	[40]	2022	C57/BL6 mice; EA.hy 926; L929; RAW264.7 cells	M@M–Ag–Sil‐MA	CD86; CD206; MPO; CitH3; CD31; CD11b; Ly‐6G;	M @ M‐Ag‐Sil‐MA hydrogel system alleviates diabetes wounds through the spatiotemporal immune regulation of macrophages and NETs
	[41]	2025	C57BL/6J mice	Ro 106‐9920	CitH3; NLRP3; IL‐1β; pro‐IL‐1β; PAD4; ASC, caspase‐1; NF‐κB	Ro106−9920 can promote diabetic wound healing by inhibiting NETs formation and blocking NLRP3 inflammasome activation
	[42]	2026	Oral mucosal defect models in both healthy rats and pigs; HUVECs; NIH/3T3 cells	PCPAB	IL‐6; TNF‐α; MPO; CitH3; IL‐1β; cfDNA	PCPAB accelerates oral wound healing by remodeling the diabetic wound microenvironment
Impaired macrophage polarization and phagocytic capacity	[43]	2025	Diabetic mouse; Pigs	OD/PBA‐PEI/Ce6@ALDA‐1 Hydrogel	ALDH2; MPO; CitH3	A Photodynamic Hydrogel Activating ALDH2 to Combat Inflammation and Enhance Angiogenesis in Diabetic Wound Healing
	[44]	2025	C57BL/6J mice; Fibroblasts; HUVECs; Keratinocytes; THP‐1	PPPS	CitH3; PAD4; CD31; CD34	PPPS accelerates diabetic wound healing by mediating NETs and M1 macrophage polarization
	[45]	2025	Raw264.7; Acs; C57BL/6N mice	NSC23766; Cl‐Ad; ML‐099; 740Y‐P; Wortmannin	MPO; CitH3; p‐PI3K, Rac1‐GTP; total Rac1	By targeting NETs and the PI3K/Rac1 signaling pathway, the phagocytic function of macrophages is effectively enhanced, thereby promoting the healing process of diabetic wounds
Inhibitor	[46]	2015	WT and *Padi4* ^*−/−*^ mice	DNase 1	PAD4; CitH3	DNase 1 accelerates wound healing in diabetic mice and normoglycemic WT mice by disrupting the NETs
	[47]	2025	T2DM patients; HSFs	MMP‐9‐IN‐1; DNase 1	MMP‐9; NE; IL‐8; CTX1; COL1	The IL‐8/MMP‐9 axis impairs wound healing in type 2 diabetes mellitus through NETs–fibroblast interaction
	[48]	2025	C57BL/6 mice; db/db mice (BKS‐Leprem2Cd479/Gpt)	P/D‐rNG‐Gel	Ly6G; CitH3	The hierarchical hydrogel coloaded with PDGF‐BB and DNase I accelerates the healing of wound models in diabetic mice
Small molecules, biomarkers, or targets	[49]	2023	(BKS‐Dock Leprem2Cd479, db/db) diabetic mice; HucMSCs; Human fibroblasts	miR‐17–5p	ROS; MPO; CitH3; CD63; CD9; TSG101	Hypo‐sEVs efficiently promoted diabetic wound healing and reduced the excessive NETs formation by transferring miR‐17–5p
	[50]	2019	DKer HMGB1 mice; HMGB1 fL/fl (Cre negative) control mice; InvEE mice;	HMGB1	CD64; Ly6G; CD45^+^; CD11b^+^; NE; CitH3;	Epithelial HMGB1 delays skin wound healing and drives tumor initiation by priming neutrophils for NETs formation
	[51]	2025	C57BL/6 mice; HSF	KIRA6	CitH3; MPO; PTGS2; ACSL4; COL I; COL III, GPX4, SLC7A11; LPO; IRE1α; p‐ IRE1α; PERK; p‐PERK; XBP1; CHOP	NETs induce ferroptosis of fibroblasts through IRE1α/ XBP1‐mediated ER stress to impair diabetic wound healing
Other drugs with therapeutic potential	[52]	2018	C57BL/6 mice; TallyHo/JngJ mice;	Alpha‐toxin (AT)	c‐IgG; AT Mab; Ly6G; CitH3; CD11b; Ly6C; F4/80;	Neutralizing alpha‐toxin accelerates healing of *Staphylococcus aureus*‐infected wounds in diabetic mice
	[6]	2023	Healthy volunteers	Insulin	ROS; MPO; CitH3; DNA; PAD4; HPRT; NE; MAPKs	Insulin improves wound healing in diabetes by regulating the formation of NET
	[53]	2018	T2DM patients;	Clarithromycin	LL‐37; IL‐17; NE; MPO	Clarithromycin may enhance the wound healing ability of T2D patients by upregulating LL‐37 on the NETs structures
	[54]	2025	C57BL/6J mice; HL‐60; HUVECs; HDFs	BQSA ‐ HCM	BMAL1; CD11b; CitH3	BQSA – HCM suppresses NETs formation and promotes diabetic wound healing via PGE2/BMAL1 pathway
	[16]	2021	Healthy subjects; T2DM patients with purulent necrotizing lesions of the lower extremities	Vitamin D3/omega‐3 PUFAs	HbA1c; AST; ESR; ALT; ROS/RHS; 25‐hydroxyvitamin D3 level; baseline blood glucose level, creatinine, urea	Reduced production of NETs and accelerated wound healing in patients with T2DM through vitamin D3/omega‐3 PUFA supplementation

*Note:* Bio‐based Hydrogel Microspheres Micro‐Cage, mPDA‐PEI@GelMA.

Abbreviations: Acs, apoptotic cells; ALDH2, aldehyde dehydrogenase 2; ALT, alanine aminotransferase; AST, aspartate aminotransferase; COL1, collagen type I; CTX1, C‐terminal cross‐linked telopeptide of type I collagen; eDNA, extracellular DNA; ESR, erythrocyte sedimentation rate; HbA1c, glycated hemoglobin level; HDMECs, human dermal microvascular endothelial cells; HMGB1, high‐mobility group box 1; HUVECs, human umbilical vein endothelial cells; LPO, lipid peroxidation; LRG1, leucine‐rich a‐2‐glycoprotein 1; MMP‐9‐IN‐1, specific inhibitor of MMP‐9; P/D‐rNG‐Gel, PDGF‐BB and DNase I co‐loaded hydrogel; pAKT, phospho‐AKT; PCPAB, positively charged protein‐based adhesive bioshield; PPPS, pine pollen polysaccharides; RHS, reactive halogen species; ROS, reactive oxygen species.

## 3. Discussion

**Figure 2:**
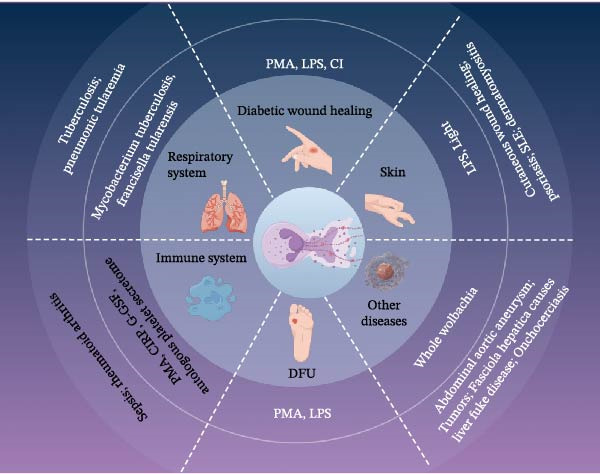
Different inducers of neutrophil extracellular trap networks and associated diseases. Figure shows a summary of the use of NETs inducers in different disease models. The center represents the core of NETs formation, radiating outward to different body systems (diabetic wound healing, DFU, respiratory system, immune system, skin, other diseases, etc.) and lists specific diseases in each system (tuberculosis in the respiratory system, sepsis and rheumatoid arthritis in the immune system, etc.).

Since the discovery of NETs, an increasing number of experts have confirmed their involvement in the pathogenesis and progression of various diseases. Although the specific mechanisms inducing and regulating NETs production have been elucidated, research in both basic experiments and clinical settings remains highly limited. As a newly identified key immune pathway and an emerging research focus, the role of NETs in wound healing progression during diabetes and the regulatory mechanisms within downstream signaling pathways following NETs formation warrant further investigation. Research into their functions under pathological conditions has become an increasingly prominent topic (Figure 2).

NETs are networks of chromatin known for their ability to defend against pathogens. However, NETs have a “double‐edged sword” role in that they can contribute to the host’s immune defense while also being implicated in the pathogenesis of various diseases, including diabetes. Current literature indicates that in many conditions, such as DM, respiratory diseases, immune system disorders, and skin diseases, the overproduction of NETs can lead to bodily damage and accelerate disease progression. Therefore, a comprehensive understanding of how neutrophils construct NETs in vitro is essential for investigating the pathogenesis of diseases associated with NETs.

In this study, we summarize the drugs and conditions used to induce NETs in vitro, the signature proteins detected, and provide an overview of recent research advances regarding the definition, characterization, and mechanisms of action of NETs. We also specifically address the process of NETs formation and its role in diabetic wound healing. From both scientific and clinical perspectives, we have established a foundation for studying the mechanisms related to NETs. Investigating the role of NETs in the progression of diabetic wound healing presents promising applications and is likely to offer new guidelines for understanding the deterioration of the disease associated with NETs formation.

Diabetic wound healing is a multifactorial and complex developmental process. According to current research on wounds in diabetic patients, the primary impact of NETs on diabetic wound healing is detrimental. However, many questions remain unanswered about how to better understand the influence of NETs on diabetic wound healing, particularly concerning the molecular mechanisms that drive this process.

The current evidence generally supports the involvement of NETs in the pathogenesis of diabetic wound healing and DFU; however, conclusions regarding “population‐level causality” should be interpreted with caution. Clinical studies have shown that levels of NETs‐associated components (such as NE, MPO, and histones) are elevated in the circulation and at the wound site of DFU patients, and are associated with infection, delayed healing, and poor prognosis, suggesting that NETs hold significant value for stratifying disease activity and prognosis. However, such studies are predominantly correlational and are susceptible to confounding factors such as the severity of infection, ischemia, debridement frequency, and antibiotic use; consequently, it remains difficult to determine whether NETs act as a “driving factor” or a “marker of association” in humans [65]. In contrast, animal models provide stronger evidence of intervention: diabetic mice exhibit elevated NETs markers (such as CitH3) and delayed wound healing, whereas PAD4 deficiency or inhibition, as well as DNase I‐mediated NETs degradation, can significantly accelerate wound healing in diabetic mice, supporting a causal relationship between NETs and impaired wound healing [32, 33]. However, it should be noted that most current models involve full‐thickness excision wounds on the backs of STZ‐ or genetically diabetic mice, which heal primarily through contraction. This differs significantly from DFUs in the weight‐bearing environment of the human foot (often accompanied by neuropathy, lower limb ischemia, repeated microtrauma, and complex microbial communities/biofilms); therefore, there are limitations to extrapolating these findings to clinical efficacy. In particular, animal models often lack the real‐world DFU microenvironment characterized by the triad of “ischemia + infection + pressure”, which may lead to an underestimation of the interaction between NETs and biofilm infections, or an overestimation of the ability of NETs inhibition alone to reverse non‐healing [165].

Furthermore, the expression of NETs in diabetes is not simply “unidirectionally enhanced”; there are “conflicting phenomena” that warrant critical comparison. Studies in patients with DFU have observed that while peripheral blood neutrophils exhibit spontaneous increases in NETosis, their capacity for inducible NETosis in response to exogenous stimuli is impaired. This suggests that diabetes may induce a “chronic pre‐excited/exhausted‐like” state: on the one hand, the continuous release of NETs sustains inflammation and tissue damage; on the other hand, the response is uncoordinated in the face of actual infectious stimuli, leading to poor infection control and prolonged inflammation [33]. Therefore, simply categorizing NETs as “harmful byproducts that should be completely suppressed” may be too simplistic; a more reasonable framework is that, while NETs offer antibacterial benefits in the early stages, their “excessive/persistent presence” in diabetic wounds—resulting from a persistently hyperglycemic and inflammatory environment—prevents the transition from the inflammatory phase to the proliferative repair phase and interferes with angiogenesis and tissue remodeling [32].

Regarding the “most harmful phase of NETs on wounds”, current evidence for direct staging remains insufficient. Both mechanistic and intervention studies suggest that the key mechanism by which NETs adversely affect wounds may occur during the window period following the inflammatory phase, when the wound should transition into the proliferative/angiogenic phase. During this period, the cytotoxic effects of NETs‐associated proteases (such as NE) and histones are more likely to inhibit granulation tissue formation, impair endothelial function, and delay re‐epithelialization [140]. However, there is currently a lack of systematic longitudinal sampling (from the same patient at different time points) to validate the causal temporal sequence of “failure of the transition from NETs burden to the healing phase”, which represents a clear gap in our understanding.

In terms of treatment, NETs‐targeted strategies (such as DNase I cleavage and PAD4 inhibition) have shown potential to promote healing in animal models; however, their risks and off‐target effects must be fully emphasized: NETs are a critical innate antimicrobial mechanism, and excessive or systemic inhibition may increase the risk of infection spread, particularly in the DFU population, which is already at high risk for infection; Upstream inhibition of PAD4 and similar targets may affect broader immune and epigenetic processes, potentially leading to unforeseen systemic effects. Therefore, a more feasible approach may involve localized, short‐term, and stratified precision interventions: for example, in patients with significantly elevated NETs markers and persistent inflammation, employing localized DNase I or phased inhibition of NETs formation in combination with standard treatments such as debridement, anti‐infective therapy, pressure relief, and revascularization, rather than long‐term systemic inhibition [32, 65].

To facilitate the clinical translation of this study, future efforts should focus on establishing a systematic research pathway. It is recommended to proceed based on verifiable hypotheses: First, conduct randomized controlled trials stratified by NETs biomarkers (such as CitH3/NE‐DNA) to evaluate the impact of NETs degradation strategies—such as the local application of DNase I—in conjunction with standard therapy on wound healing time, infection recurrence, and amputation rates, while rigorously monitoring safety. This approach will not only validate the causal role of NETs in DFU but also reveal their potential for clinical application. Second, through longitudinal dynamic sampling, determine whether NETs exert a persistent detrimental effect during the critical transition phase from inflammation to proliferation, thereby defining the optimal timing and duration of precise interventions to balance the needs for antimicrobial action and tissue repair. To enhance the translational value of research, it is necessary to establish DFU models that simultaneously integrate complex factors such as ischemia, neuropathy, pressure load, and biofilm infection, and validate these findings in human samples using spatio‐omics and immunohistochemistry. At the mechanistic level, we should thoroughly analyze the relative contributions of “excessive NETs production” and “insufficient NETs clearance” in diabetes, particularly by directly investigating whether specific barriers to NETs clearance exist in diabetic conditions. Concurrently, we need to further elucidate the specific cellular interaction mechanisms through which NETs influence angiogenesis and endothelial cell phenotypes (such as EndMT), and explore the possibility of targeting relevant pathways to improve healing without significantly compromising host defense capabilities.

## 4. Conclusion

In this review, we present a comprehensive systematic analysis of the commonly used inducers of NETs in diabetic wound healing, including pathogens, inflammatory mediators, and chemical triggers. We also describe the various pathways through which NETs are formed. Additionally, we summarize the findings of recent experimental and clinical studies on NETs in the context of diabetic wound healing. The data presented in this paper clearly indicate that neutrophils generate a diverse array of NETs during the healing process in diabetes. Consequently, we hypothesize that NETs may play a crucial role in diabetic wound healing. Furthermore, we discuss the mechanisms by which NETs contribute to wound healing, noting that their presence at the wound site is closely associated with delayed healing. We emphasize the development and significance of NETs in diabetic wound healing, particularly in relation to DFU. In summary, NETs represent a potential therapeutic target and an important component in the process of diabetic wound healing, warranting further investigation.

At present, there are still many unknowns about the interaction between NETs and the diabetic microenvironment, and it is crucial to dig deeper into the specific receptors and their downstream signaling pathways of NETs to comprehensively reveal the specific mechanism of NETs in the pathological process of diabetes. At the same time, the development of a composite model of “diabetes co‐infection” cannot be ignored, which helps to simulate real clinical scenarios to the greatest extent and build a more clinically relevant experimental model for research. At the level of clinical translation, we hope that basic research results can be efficiently translated into practical applications, accurately grasp the best time for individualized treatment, and improve treatment effects. In addition, for example, it is also necessary to systematically evaluate the long‐term safety of DNase gel applied locally, which is an important link to ensure the safety and reliability of interventions in clinical application. Given that NETs are a key target for the treatment of DFU, their intervention strategies must balance efficacy and safety. Therefore, we emphasize precise intervention, which involves taking specific treatment measures for different stages of the disease and prioritizing local administration to reduce systemic side effects. Looking forward to the future, research should further focus on interdisciplinary integration, organically integrating immunology, material science, metabolomics, and other multi‐disciplinary knowledge, so as to form a comprehensive and in‐depth understanding of NETs in diabetes and its complications, and ultimately promote a comprehensive breakthrough from basic research to clinical application.

## Author Contributions

Jiaojiao Xue is responsible for writing, the conception of the article, drawing, document sorting, sorting out the ideas of the article. Zeyu Wang and Wenxiu Qi are responsible for controlling the overall quality of the article and revising the article.

## Funding

This work was supported by “Noncommunicable Chronic Diseases‐National Science and Technology Major Project” (No. 2023ZD0509300), Medical Center Project of Changchun University of Traditional Chinese Medicine Affiliated Hospital (DXZX‐02‐02; DXZX‐04‐08), Innovation Team and Talents Cultivation Program of National Administration of Traditional Chinese Medicine (No. ZYYCXTD‐D‐202001) and Jilin Province Science and Technology Development Plan Project “Jilin Province Traditional Chinese Medicine Respiratory Disease Clinical Medical Research Center” (YDZJ202202CXJD049).

## Conflicts of Interest

The authors declare no conflicts of interest.

## Data Availability

The data that support the findings of this study are available from the corresponding author upon reasonable request.
